# Advancing AI and data science for health in Africa: education, collaboration, and applications for global health priorities

**DOI:** 10.3389/fpubh.2026.1820671

**Published:** 2026-05-11

**Authors:** Saloshni Naidoo, Cheng He, Henry Mwambi, Heather Mattie, Nisha Nadesan-Reddy, David Guwatudde, Candida Moshiro, Chris Guure, Angela Chukwuu, Ina Danquah, Isabel Madzorera, Sandra Barteit, Onisimo Mutanga, Mosa Moshabela, Till Bärnighausen, Wafaie Fawzi

**Affiliations:** 1 Department of Public Health Medicine, University of KwaZulu Natal, Durban, South Africa; 2 Department of Global Health and Population, Harvard T.H. Chan School of Public Health, Boston, MA, United States; 3 Discipline of Statistics, School of Agriculture and Science, University of KwaZulu-Natal, Pietermaritzburg, South Africa; 4 Department of Biostatistics, Harvard T.H. Chan School of Public Health, Boston, MA, United States; 5 Department of Epidemiology and Biostatistics, Makerere University, Kampala, Uganda; 6 Department of Epidemiology and Biostatistics, School of Public Health and Social Sciences, Muhimbili University of Health and Allied Sciences, Dar es Salaam, Tanzania; 7 Department of Biostatistics, School of Public Health, University of Ghana, Accra, Ghana; 8 Department of Statistics, University of Ibadan, Ibadan, Nigeria; 9 Center for Development Research (ZEF), University of Bonn, Bonn, Germany; 10 Division of Community Health Sciences, School of Public Health, University of California, Berkeley, Berkeley, CA, United States; 11 Heidelberg Institute of Global Health (HIGH), Faculty of Medicine and University Hospital, Heidelberg University, Heidelberg, Germany; 12 School of Agriculture and Science, Discipline of Geography, University of KwaZulu-Natal, Pietermaritzburg, South Africa; 13 School of Nursing and Public Health, University of KwaZulu-Natal, Durban, South Africa; 14 University of Cape Town, Cape Town, South Africa; 15 Africa Health Research Institute (AHRI), KwaZulu-Natal, South Africa; 16 Harvard Center for Population and Development Studies, Cambridge, MA, United States; 17 Department of Epidemiology, Harvard T.H. Chan School of Public Health, Boston, MA, United States; 18 Department of Nutrition, Harvard T.H. Chan School of Public Health, Boston, MA, United States

**Keywords:** Africa, climate change, data science, health education, WASHA Takwimu

## Abstract

**Background:**

Africa faces a shortage of health data scientists. Despite bearing 25% of the global disease burden, it has only 3% of the world’s healthcare workforce and even less health data science expertise. As artificial intelligence and data science transform global healthcare, from disease surveillance to precision medicine, this capacity gap poses a significant threat to Africa’s ability to address its health challenges and harness its growing young population for health innovation.

**Methods:**

We describe the WASHA Takwimu program, a multi-institutional capacity-building initiative funded by the National Institutes of Health (NIH) Data Science Initiative for Africa (DSI Africa) consortium. Operating through a hub-and-spoke model anchored by the University of KwaZulu-Natal (UKZN), Harvard T. H. Chan School of Public Health, and Heidelberg Institute of Global Health, the project has spoke partners in Ghana, Nigeria, Tanzania, and Uganda. The program delivers training through multiple modalities: master’s degrees, postdoctoral fellowships, short courses, and professional development activities. The curriculum integrates data science methods with applications in global health priority domains, including health systems strengthening and food systems, climate change, and planetary health, using competency-based, application-focused, and digitally enhanced approaches.

**Results:**

From 2020 to 2024, WASHA Takwimu (Kiswahili for “Ignite Data”) trained postdoctoral fellows, doctoral students, and other early-career researchers and practitioners across five African countries. The program has supported the development of a Master of Health Data Science program at UKZN and contributed to faculty capacity and curriculum development for a similar program at Makerere University. Key achievements include successful faculty exchanges replacing costly international student placements, integration of technological innovations in learning delivery, and strategic partnerships with national research and policy organizations which connect training to policy-relevant applications. Critical lessons have been learned regarding infrastructure constraints, data governance challenges, gender inequality in participation, and the importance of managing student expectations while maintaining rigorous entry requirements.

**Implications:**

WASHA Takwimu demonstrates that network-based approaches combining graduate training, faculty development, and stakeholder engagement can build sustainable health data science capacity in Africa. The program’s hub-and-spoke model offers a replicable framework balancing centralized coordination with distributed implementation, while the Master’s programs provide adaptable templates for building similar educational offerings at other African institutions. However, consolidating current achievements must precede ambitious expansion. Strategic priorities include strengthening partnerships with national research councils and Ministries of Health, addressing persistent gender disparities, deepening private sector engagement, and progressively developing PhD programs. As Africa’s population approaches 2.5 billion by 2050, investments in health data science capacity will prove essential for addressing continental health priorities and positioning African institutions as global leaders.

## Background

Artificial intelligence (AI) and data science are driving a transformative era in public health and healthcare globally, fundamentally altering disease prevention, public health surveillance, clinical diagnostics, treatment, and health system operations (1). By leveraging machine learning algorithms, data analytics, and automation, AI has the potential to enhance medical decision-making and diagnosis, and improve treatment outcomes, while also boost productivity, improve care quality, and reduce costs (2). The global AI healthcare market is experiencing unprecedented growth, projected to expand from $11.2 billion in 2023 to $427.5 billion by 2032 (1), reflecting the increasing recognition of data-driven approaches as essential to modern healthcare delivery. However, this transformation is highly uneven, and its implications vary across regions. While regions facing severe health system constraints stand to gain the most, they often possess the least capacity to adopt and shape these technologies.

Africa’s population currently stands at approximately 1.5 billion people, about 19% of the global population (3). With projections of the African population reaching 2.5 billion by 2050, approximately 25% of the world’s population (3), the continent faces both opportunities and challenges in health service delivery and data infrastructure (4). The continent faces a high burden of disease, exemplified by a maternal mortality ratio of 531 per 100,000 live births, more than double the global average of approximately 223 per 100,000, and a 31% prevalence of childhood stunting, compared to global average of around 22%, alongside persistent constraints in the health workforce and research capacity (4). This profound disparity stems from limited infrastructure, a lack of trained personnel, insufficient funding, brain drain of trained healthcare workers to higher-income countries, and economic instability, all of which hinder access to quality health services (4). Furthermore, critical gaps exist in applying data science to the complex interactions between climate change, food systems, and planetary health. Despite the continent’s vulnerability to environmental shifts, the capacity to model how these factors drive food insecurity and poor nutritional outcomes remains underdeveloped. At the same time, Africa’s rapidly growing and youthful population presents an opportunity: if equipped with advanced quantitative and computational skills, a new generation of African researchers and practitioners could drive locally relevant innovation in digital health and health analytics. However, the region currently has the lowest capacity to develop and deploy advanced AI and data science solutions compared with other parts of the world, raising concerns that the benefits of data-driven health systems may widen existing inequities.

Data science, including generative AI (genAI), is particularly relevant for African settings because it can address problems shaped by fragmented systems, sub-optimal food systems and environmental vulnerability. Examples include linking patient information across disconnected facilities to support continuity of care in fragmented health systems (5), integrating satellite imagery and climate data to anticipate disease risk, food insecurity and nutritional emergencies and guide preparedness in remote areas (6), and optimizing supply chains (including vaccine cold chains) where transport constraints and unreliable electricity challenge service delivery (7). Despite growing demand and an expanding number of postgraduate programs and collaborative initiatives, major barriers continue to limit progress in enhancing the use of data science in Africa. Data science training programs have expanded across the continent in recent years (Table 1); however, most programs remain general in scope, with limited focus on health-specific applications. First, driven by a lack of domestic investment and public sector prioritization, infrastructure constraints, such as uneven internet connectivity, power interruptions, limited computing resources, and the high cost of proprietary software, restrict both training and real-world implementation (8). Second, limitations in data quality, interoperability, access, and governance, alongside underdeveloped frameworks for privacy protection, ethical use, and responsible data sharing, constrain the use of routine health information and emerging data sources (4, 9, 10). Third, shortages of faculty expertise and persistent gender disparities limit the development of a sustainable and representative pipeline of health data scientists and academic leaders (8, 11).

**Table 1:** Data Science training programs by country and university offering in Africa in 2025.

Country	University	Program(s)
Nigeria	Pan-Atlantic University (Lagos)	MSc in Data Science
Uganda	Makerere University	MSc and PhD in Data Science (under-going institutional review for approval)
South Africa	University of Johannesburg	MSc of Applied Data Science; DPhil in Data Science
	University of Cape Town	MSc in Data Science
	University of Pretoria	MSc in Advanced Data Analytics
	University of the Witwatersrand	MSc in Data Science, Postgraduate Diploma in Data Science
	University of KwaZulu-Natal	MSc in Data Science, Postgraduate Diploma in Data Science
Kenya	Taita Taveta University	MSc Data Science and Analytics
	University of Nairobi	MSc in Data Science
Rwanda	University of Rwanda (ACE-DS)	MSc and PhD in Data Science
Botswana	Botswana International University of Science and Technology	MSc in Info Systems and Data Management

Addressing these barriers requires coordinated capacity-building that goes beyond short-term individual training. A practical approach involves (i) training graduates with integrated competencies spanning statistics, computing, and health and food systems domain expertise; (ii) strengthening faculty capacity through exchanges, short courses, and mentorship to keep pace with rapidly evolving methods; and (iii) ensuring institutional and ecosystem readiness through partnerships that improve infrastructure, enable access to high-quality datasets, and create career pathways in academia, government, and industry (8, 12). In this context, we present the WASHA Takwimu (Kiswahili for “Ignite Data”) program as a case of a network-based, hub-and-spoke model for building sustainable health data science capacity in Africa (4, 8, 12). Here, we aim to describe its training modalities and partnerships, the development of master’s programs at African institutions, lessons learned, and actionable recommendations for replication and scale-up. In this context, WASHA Takwimu was established in 2020 as part of the NIH Data Science Initiative for Africa (DS-I Africa). In its initial phase, the program focused on training early-career researchers and public health professionals through short courses, fellowships, and collaborative research activities. Over time, this approach evolved toward a more institutionalized model of capacity building, recognizing the need for sustainable training pathways embedded within African universities.

## WASHA Takwimu program overview

**Figure 1:**
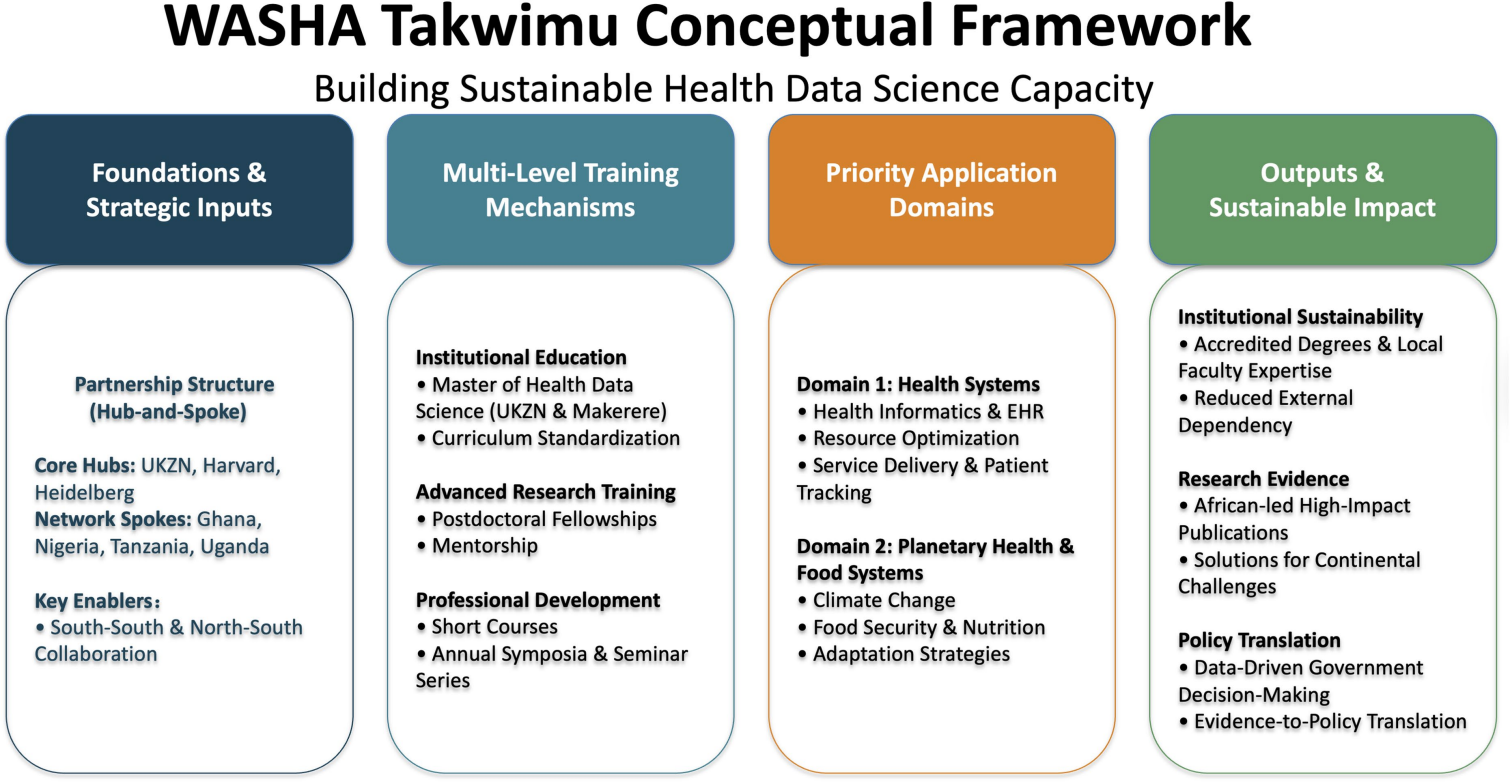
WASHA Takwimu program framework.

Building on the established Africa Research, Implementation Science and Education (ARISE) Network, a multi-country partnership of leading African universities founded in 2014, the WASHA Takwimu program operates through a hub-and-spoke model anchored by three primary institutions: the University of KwaZulu-Natal (UKZN) in South Africa, Harvard T. H. Chan School of Public Health (HSPH), and the Heidelberg Institute of Global Health (HIGH). The model extends through spoke partnerships with universities in Ghana, Nigeria, Tanzania, and Uganda, creating a comprehensive network spanning multiple African regions (Figure 1). This institutional arrangement facilitates resource sharing while maintaining local autonomy, balancing centralized coordination with distributed implementation. The program’s design operationalizes South–South and North–South collaboration frameworks, prioritizing sustainable institutional development over individual training alone.

The program’s long-term goal is to develop advanced data-skilled researchers in health data science in Africa through a rigorous curriculum and a set of training and research activities designed to address the health needs of the continent. The program pursues three interconnected aims designed to create sustainable capacity across multiple levels of the health research ecosystem:

1. Train mid-level and senior researchers at UKZN in South Africa to work as educators and principal investigators leading independent research programs focused on using data science to address important global health challenges of national, regional, and global relevance.
2. Build a critical mass of junior public health and medical professionals across South Africa and four other countries in Africa in the ARISE Network who can design and successfully carry out rigorous health research projects using data science through short-course training with professional development activities.
3. Develop and institute sustainable master’s degree programs in health data science at African institutions to serve the research training needs in the African region, while building a culture of collaborative training across the WASHA Takwimu network and beyond.

The WASHA Takwimu curriculum integrates data science methods with applications in two global health priority areas selected for their critical importance to Africa:

- Health Systems Strengthening: In health systems, data science enables addressing Africa-specific challenges such as predicting healthcare needs and optimizing allocation of scarce resources and improving service delivery in resource-constrained settings. Students learn to work with routine health information systems, population surveys, and extracting and integrating data from diverse sources including paper-based records and electronic patient records.
- Food Systems, Climate Change, and Planetary Health: In this domain, training addresses the bidirectional relationship between climate change and health outcomes through food systems. Students learn to integrate satellite imagery, climate data, agricultural production data, nutrition surveys and epidemiologic constructs to understand how climate variability affects food security, nutrition and population health.

## Training components

The program delivers capacity building through multiple interconnected training modalities, each addressing different career stages and learning needs:

1. Training Master’s Students: Master’s training provides specialized coursework in biostatistics, machine learning, and health informatics, with thesis projects solving region-specific problems under faculty and industry mentorship;
2. Postdoctoral Fellowships: Postdoctoral fellowships combine advanced research in AI-based diagnostics, sustainable food systems modeling, and genomic epidemiology with professional development in grant writing, teaching, and scientific communication, preparing fellows for faculty or leadership positions;
3. Short Courses: Short courses offer intensive training in predictive modeling, health data visualization, and ethical AI for mid-career professionals, enabling ministries of health, NGOs, and hospitals to develop in-house expertise without requiring full degree programs;
4. Annual Symposia: Annual symposia bring together trainees, faculty, policymakers, and industry partners to showcase research, discuss emerging trends, and forge collaborations through presentations and professional development workshops;
5. Monthly Seminar Series: Monthly seminars maintain momentum through technical presentations and broader discussions by faculty, external experts, and trainees, with recorded content archived online for asynchronous learning and ongoing community engagement.

## Developing a master’s in health data science for Africa

**Figure 2:**
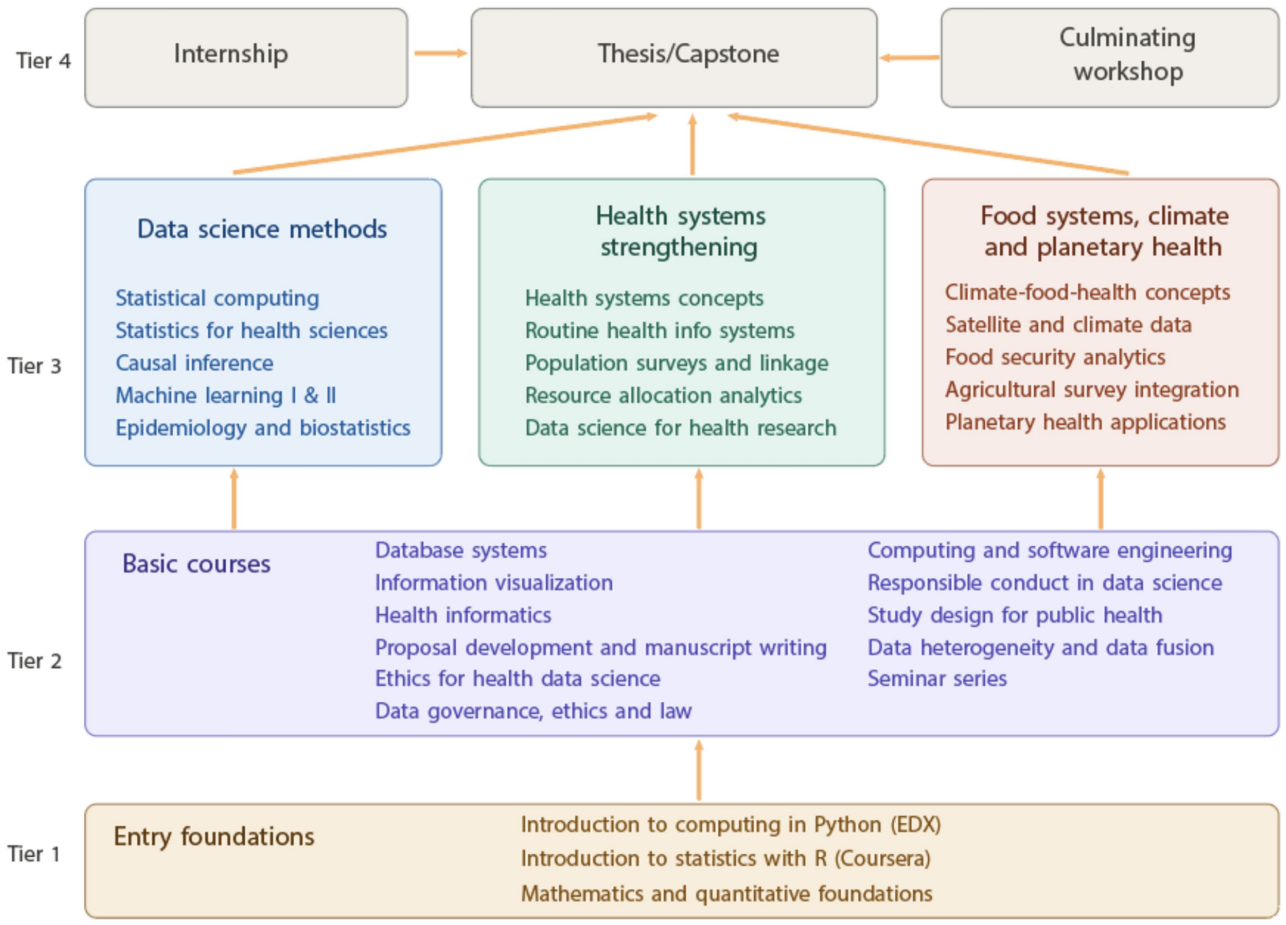
Unified competency and curriculum framework for the master of health data science. This diagram illustrates the pedagogical progression and core components adopted by the WASHA Takwimu network. The learning trajectory progresses from Foundations and Basic Courses (bottom) to specialized Data Science Methods and Domain Applications (top), culminating in the Internship and Thesis. Specific timelines and scheduling are adapted to align with each institution’s academic calendar.

A critical component of WASHA Takwimu’s sustainability strategy is the establishment of accredited Master of Health Data Science (MHDS) programs. Two institutions have taken the lead in developing comprehensive programs: UKZN in South Africa and Makerere University in Uganda. These programs are structured around a unified framework (Figure 2). While both programs share the common goal of building health data science capacity through specialized training, they are exhibiting distinct approaches reflecting their institutional contexts and national regulatory frameworks:

1. *Program structures and credit systems*. The proposed UKZN program, developed as an initiative of the NIH-funded WASHA Takwimu training grant, is structured as a 192-credit qualification at National Qualifications Framework (NQF) Level 9 (master’s degree level in South Africa’s national framework), delivered over four semesters through a blended format. The program comprises compulsory modules, elective modules and a research dissertation, following South Africa’s Higher Education Qualifications Sub-Framework requirement of 10 notional hours per credit.

The proposed Makerere’s 2-year program uses a credit system aligned with East African frameworks, structured to establish foundational competencies in Year 1 and advance specialized applications in Year 2. Students culminate their training with a Plan B project, which offers greater flexibility than traditional dissertations, allowing diverse research outputs aligned with career trajectories and local health priorities.

2. *Core competencies and curriculum emphases*. The proposed UKZN’s curriculum covers six key domains: (1) statistical foundations where participants develop biostatistics expertise necessary for health data science; (2) computing and software engineering where students become competent in programming and database management; (3) artificial intelligence covering model selection, training, evaluation, and interpretation for clinical prediction, classification, and clustering; (4) epidemiology providing understanding of disease patterns, risk factors, and intervention impacts to enhance health data analysis capabilities; (5) health systems where students learn to integrate data from routine health information systems and population surveys; (6) food systems, climate change and planetary health providing understanding of how climate change impacts food security and public health, and how data science can develop sustainable solutions.

The proposed Makerere’s program develops six competencies with different emphases: (1) ability to acquire and pre-process data for health data science analytics; (2) ability to recognize study designs and their application in public health contexts; (3) ability to understand data heterogeneity (variability in data types, formats, and sources) and innovatively merge structured and unstructured data; (4) ability to use data visualization tools and communicate results to multidisciplinary audiences; (5) ability to evaluate modeling contexts and apply statistical and machine learning approaches to analyze health-related data; (6) ability to use programming languages in data science; and (7) knowledge of ethical standards in health research and data science with ability to apply these in practice.

3. *Pedagogical approaches and specialization options*. The proposed UKZN program offers three major elective tracks allowing deeper specialization in specific domains: Health Systems Strengthening, Food Systems/Climate Change/Planetary Health, and Data Governance/Ethics/Law. This structure explicitly integrates two specialized domain areas (health systems and climate/food systems) as distinct tracks, reflecting South Africa’s national priorities on planetary health. UKZN intends to include a full 16-credit elective on data governance, ethics, and law, with emphasis on African data protection legislation and FAIR principles, ensuring graduates understand responsible data stewardship in African contexts.

Makerere integrates domain topics across required courses with less emphasis on electives, ensuring all graduates receive broad exposure to health systems, epidemiology, and ethics through compulsory modules. Makerere’s inclusion of machine learning as a standalone course prepares graduates to deploy and maintain models in production environments. Ethics is addressed through a 3-credit required course on Responsible and Ethical Conduct in Data Science, making it compulsory for all students rather than an elective option. The two-semester seminar series integrated throughout Makerere’s Year 2 provides ongoing opportunities for students to present their work, receive peer and faculty feedback, and engage with current research in health data science, creating a scholarly community among students.

4.*Development processes and quality assurance*. UKZN’s ongoing development involves a comprehensive analysis of international health data science programs, reviewing curricula from institutions including Harvard and HIGH to identify best practices and credit distributions. Stakeholder workshops engaged participants from spoke partner universities in Ghana, Nigeria, Tanzania, and Uganda, as well as UKZN faculty, ensuring the program meets local needs across the WASHA Takwimu network. The UKZN program is currently in an advanced stage of development. While the core curriculum has been largely finalized, it is undergoing the university’s quality assurance and accreditation processes, with ongoing refinements based on feedback.

Makerere is developing its program fully funded with the university’s internal resources, demonstrating strong institutional commitment to health data science capacity building, independent of external grant funding. The development involved faculty collaboration across the School of Public Health and consultation with stakeholders in Uganda’s health sector, including the Ministry of Health and international health organizations operating in the country. The curriculum underwent iterative refinement based on feedback from potential employers to ensure alignment with national health data priorities and to create pathways for graduate employment within Uganda’s health system. Makerere’s curriculum is currently undergoing review by the relevant internal departments, following the university’s established protocols for postgraduate programs. The first cohort of trainees is expected to be enrolled in the academic year 2026–2027, following completion of Makerere’s internal approval processes, and by the country’s National Council for Higher Education.

5. *Distinctive strengths and complementary models*. These two programs offer complementary models for African health data science education, each with distinctive strengths suited to different institutional contexts. At UKZN, two elective streams are offered, each aligned with one of the program’s two domains: planetary health and climate change. A more comprehensive dissertation component requiring substantial independent research (96 credits representing half of the total program), and formal integration with international quality standards through NQF Level 9 designation. This approach offers a blueprint for institutions in countries with rigorous national qualification frameworks and research-intensive master’s traditions.

Makerere’s model demonstrates a more agile curriculum with emerging technical topics featured as standalone courses rather than integrated modules, a flexible project-based capstone allowing diverse research outputs aligned with various career paths in academia, government, NGOs, or the private sector, and sustainable development using internal university resources without dependence on external grant funding. This approach shows how universities can develop innovative programs independently while maintaining curricular responsiveness to evolving data science practice.

In addition to technical and methodological training, both programs explicitly prioritize the development of interdisciplinary and transdisciplinary competencies, recognizing that health data science is inherently collaborative and embedded within complex sociotechnical systems. Trainees are expected to work across disciplinary boundaries and engage with a wide range of stakeholders, including clinicians, public health practitioners, policymakers, and technical experts. These skills are developed through collaborative project-based learning, the use of real-world datasets, mentorship across disciplines, and structured engagement with policy and implementation partners. By integrating these elements into the curriculum, the programs aim to produce graduates who are not only technically proficient but also capable of translating data-driven insights into practice within diverse health system contexts.

## Platform for network-wide adoption

These two MHDS programs serve as foundational templates for other WASHA Takwimu partner institutions seeking to develop their own health data science programs. This platform approach aims to offer several advantages: (1) Reduced development time and costs as partners can build upon proven curricula rather than starting from scratch; (2) Harmonized competencies across the network ensuring graduates meet consistent standards while allowing contextual adaptations; (3) Shared resources including teaching materials, case studies, and assessment tools developed by UKZN and Makerere; (4) Collaborative quality assurance where partner institutions can learn from each other’s accreditation experiences and provide mutual external review; and (5) Faculty exchange for capacity strengthening, enabling experienced faculty from established programs to support partner institutions during critical early launch phases through collaborative teaching, mentorship, and implementation guidance across sub-Saharan Africa.

## Lessons learned

The implementation of WASHA Takwimu has yielded valuable insights into building sustainable health data science capacity in Africa. Several important lessons inform both the continued development of the program and provide guidance for similar initiatives across the continent.

### Value of network-based partnerships

The hub-and-spoke model has proven highly effective in balancing centralized coordination with distributed implementation. By building up the network through UKZN, HSPH and HIGH, while extending through spoke partnerships in Ghana, Nigeria, Tanzania, and Uganda, WASHA Takwimu has achieved scale while maintaining contextual relevance. This network structure enables resource sharing, facilitates faculty exchanges, and creates opportunities for cross-institutional mentorship that would be difficult to achieve through isolated bilateral partnerships. The network has also facilitated bidirectional exchange between African and international partners. For example, faculty from HSPH and Heidelberg have participated in in-person short courses delivered at African partner institutions, including recent training activities in 2024, where joint teaching teams conducted intensive courses locally. These exchanges reinforce the circularity of the program, moving beyond unidirectional training toward reciprocal knowledge sharing and collaborative capacity building. The network has fostered South–South collaboration, with African institutions increasingly sharing curricula, teaching materials, and best practices directly with one another, reducing dependency on Northern partners over time.

### Engagement with national and regional organizations

Collaboration with national research and policy organizations has been an important component of the program. For example, engagement with institutions such as the South African Medical Research Council (SAMRC) and other national stakeholders has supported access to inform the design of training activities, and created opportunities to align research outputs with policy priorities. These interactions have also provided platforms for trainees to engage with real-world policy questions and implementation challenges. Such experiences highlight the importance of embedding capacity-building initiatives within existing national research and health systems, rather than operating as parallel structures.

### Integration of technological advancements in training

The program has successfully integrated modern pedagogical approaches with technological innovations to enhance accessibility and learning outcomes. The shift to blended learning models, combining face-to-face workshops with online-enhanced delivery, has proven particularly valuable. The monthly seminar series, conducted virtually, maintains momentum between annual symposia while recorded content provides asynchronous learning opportunities for those unable to attend live sessions. The use of cloud-based computing platforms has partially mitigated infrastructure limitations, allowing students in resource-constrained settings to access computational resources for data science training.

### Scalability and sustainability considerations

Achieving sustainability requires moving beyond dependence on external grant funding. The development of master’s programs at UKZN and Makerere University represents a critical step toward institutionalizing health data science training within African universities. These programs, once approved, will generate tuition revenue and secure permanent faculty positions, ensuring continuity beyond the initial grant period. The platform approach, where UKZN and Makerere University curricula serve as templates for other institutions, accelerates program development and reduces costs for spoke partners. However, sustainability also requires continued engagement with private sector partners who can provide scholarships, internships, and employment opportunities for graduates. Public-private partnerships remain essential for long-term viability; therefore, efforts toward this need to be continuously pursued.

### Challenges of training abroad and program modifications

The program initially envisioned training African students at HSPH and HIGH, but experience revealed significant practical and strategic challenges with this approach. High costs associated with travel and living expenses in Boston and Heidelberg made this model financially unsustainable at scale. These challenges prompted a strategic shift toward faculty exchanges rather than extended student placements abroad. African faculty now spend shorter, targeted periods at HSPH developing curricula, refining teaching practices, and establishing research partnerships. However, the program has seen encouraging counter-examples, with students and researchers returning to their home institutions, obtaining promotions, and actively contributing to health data science capacity building in their countries. This approach multiplies impact: returning faculty train hundreds of students at their home institutions, adapting international materials to African contexts using local datasets. The model has generated positive feedback and appears more sustainable both financially and in terms of retaining talent within Africa.

### Managing expectations and student preparedness

Experience has revealed mismatches between student expectations and what the grant can realistically provide. Some applicants anticipated full funding for extended international training, while the program prioritizes shorter, intensive training experiences and local capacity development. Clear communication about program scope and available support has been essential to managing these expectations. Additionally, not all applicants possess the prerequisite skills for master’s and postdoctoral training in health data science. The program requires strong foundations in mathematics, statistics, and increasingly, programming. Identifying candidates with adequate preparation while remaining inclusive to underrepresented groups requires careful balancing. The program has addressed this through preparatory short courses and by clearly articulating entry requirements, though recruitment of adequately prepared candidates remains an ongoing challenge, particularly for postdoctoral positions. In addition, the program initially envisioned training African master’s students at HSPH but shifted to faculty exchanges for greater systemic impact. This approach multiplies reach: returning faculty train hundreds of students at home institutions. The model has generated positive feedback, with participants tailoring Harvard materials to African contexts using local datasets.

In addition to academic preparedness, issues of equity and access remain important considerations. The program has sought to promote inclusivity by engaging participants from multiple countries within the network and by offering short courses and blended learning opportunities that reduce barriers to participation. While dedicated funding for full scholarships remains limited, efforts have been made to support trainees through travel support, access to shared resources, and mentorship. Addressing the needs of underrepresented groups, including individuals from resource-constrained settings, remains a priority, and further work is needed to expand financial support mechanisms and broaden access to training opportunities.

### Recommendations

Building on the lessons learned from the WASHA Takwimu program, several priorities emerge for strengthening health data science capacity in Africa. First, capacity-building efforts should continue to prioritize network-based models that enable resource sharing and sustained collaboration across institutions. Second, greater alignment with national research bodies is essential to ensure that training remains responsive to policy needs and creates clear pathways for graduate employment. Third, sustained investment in faculty development and institutional programs, including Master’s and future PhD training, is critical for long-term sustainability. Finally, expanding access through targeted support for underrepresented groups will be important to ensure both inclusivity and relevance to real-world applications.

In addition, there is growing opportunity to integrate emerging areas such as Natural Language Processing into health data science training. Incorporating practical components that involve the analysis of policy and institutional documents could enhance students’ ability to engage with unstructured data while generating insights relevant to public health decision-making.

## Conclusion

WASHA Takwimu demonstrates that network-based approaches can effectively address Africa’s critical shortage of health data scientists while building sustainable capacity. The program has provided short-term and long-term training through fellowships and short-courses provided to postdoctoral fellows, doctoral students, and other early-career researchers and practitioners, across five African countries and establishing foundational Master of Health Data Science programs at UKZN, and contributing to capacity development for the establishment of a similar program at Makerere University. These achievements illustrate how coordinated investments in graduate training, faculty development, and stakeholder engagement can create systemic change beyond isolated individual capacity building.

As the program advances, consolidating current developments must take precedence over ambitious expansion. The master’s programs at UKZN and Makerere University are still undergoing accreditation processes while the program at UKZN is still in earlier stages of development. Ensuring these foundational programs achieve full accreditation, successfully produce graduates, and establish robust quality assurance mechanisms is essential before the wide dissemination of program templates. Such an approach prioritizes long-term quality and sustainability over premature scaling that could dilute impact. Strategic regional expansion should leverage existing partnerships, particularly the ARISE Network, which provides an established platform for rollout across Ghana, Nigeria, Tanzania, and Uganda. Spoke institutions can progressively adapt UKZN and Makerere University curricula to their national regulatory frameworks and local health priorities, beginning with short courses and graduate certificates before advancing to full degree programs. Critical to this expansion is systematic engagement with policy organizations. The program’s cooperation with national research councils, ministries of health, and regional bodies such as Africa CDC will ensure that training addresses priority health challenges, creates employment pathways for graduates, facilitates access to essential datasets, and establishes channels for translating research findings into policy action. These policy partnerships serve as key enablers for sustainable impact at scale.

As the Africa’s population grows toward 2.5 billion by 2050, comprising a quarter of humanity, investments in health data science capacity will prove critical to addressing Africa’s unique health challenges and shaping global health outcomes. WASHA Takwimu has established strong foundations through innovative training models, replicable curricula, and dynamic networks. By consolidating these achievements, expanding strategically, and maintaining a focus on sustainability and equity, the program positions African institutions not as recipients of external expertise, but as leaders and contributors in global health data science. This approach enables them to conduct research that addresses continental priorities and ultimately improves health outcomes for all Africans.
